# Supplementary material for: Online Home-Based Physical Activity Counteracts Changes of Redox-Status Biomarkers and Fitness Profiles during Treatment Programs in Postsurgery Female Breast Cancer Patients
Source: Antioxidants (Basel). 2023 May 22;12(5):1138. doi: 10.3390/antiox12051138 (PMC10215480; doi:10.3390/antiox12051138)
Supplement: Supplementary file 1 [file antioxidants-12-01138-s001.zip › antioxidants-2378174-supplementary-1.pdf]

Table S1. Sequence of primers used for RT-qPCR analysis.

Target gene	Forward	Reverse
Cyclophilin A	5’-GTCAACCCCACGGTGTTCTT-3’	5’-CTGCTGTCTTTGGGACCTTGT -3’
HSP70	5’-AGGGGCCTTTCCAAGATTGC-3’	5’-GCAAACACAGGAAATTGAGAACT-3’
HSP27	5’-GCAGGACGAACATGGCTACAT-3’	5’-TGGAGGCAGCGTGTATTTCC-3’
SOD1	5’-AAAGATGGTGTGGCCGATGT-3’	5’-GCCAATGATGCAATGGTCTCC-3’
SOD2	5’-CCCTGGAACCTCACATCAAC-3’	5’-GGTGACGTTCAGGTTGTTCA-3’
GPX1	5’-ACGATGTTGCCTGGAACTTT-3’	5’-TCGATGTCAATGGTCTGGAA-3’
